# Editorial: Advances in Analytical Methods for Drugs of Abuse Testing

**DOI:** 10.3389/fchem.2019.00589

**Published:** 2019-08-21

**Authors:** Shanlin Fu, Christophe Stove, Simon Elliott

**Affiliations:** ^1^Centre for Forensic Science, University of Technology Sydney, Ultimo, NSW, Australia; ^2^Laboratory of Toxicology, Department of Bioanalysis, Ghent University, Ghent, Belgium; ^3^Elliott Forensic Consulting, Birmingham, United Kingdom; ^4^Department of Analytical, Environmental and Forensic Sciences, King's College London, London, United Kingdom

**Keywords:** drug testing, drugs of abuse, new psychoactive substances, synthetic opioids, fentanyl analogs, synthetic cannabinoid receptor agonists, metabolomics, HRMS

This collection focuses on innovative approaches and analytical advancement in drugs of abuse testing and monitoring. Substance abuse negatively impacts all facets of society. Drug testing programs have been developed in many jurisdictions to service law enforcement agencies for drug intelligence, criminal justice systems for prosecution of drug-related crimes, and the health industry for harm minimization.

One significant challenge in drugs of abuse testing is the continuous emergence of new psychoactive substances (NPS) in the illicit drug market. Routine methods of analysis are no longer effective in screening NPS due to the lack of structural information and commercial reference materials which are needed for performing targeted analysis of these NPS. Scientists are developing non-targeted approaches to overcome this difficulty. In this regard, the use of high-resolution mass spectrometry (HRMS) operated in data dependent acquisition (DDA) mode or more often data independent acquisition (DIA) mode has drawn a wide interest among forensic toxicologists and chemists to advance the non-targeted approach for NPS detection (Pasin et al., 2017). In this topic collection, Klingberg et al. investigated the mass fragmentation during the collision-induced dissociation process of a series of synthetic opioids including fentanyl derivatives, AH series opioids, U series opioids, W series opioids and MT-45, and identified a number of common fragments that can potentially be used as markers to indicate the presence of a particular synthetic opioid class of compounds within a sample.

Another significant advancement in the area of non-targeted analysis is the development of activity-based assays such as the one for the detection of synthetic cannabinoid receptor agonists (SCRAs) based on interactions of the compounds with CB_1_ and CB_2_ receptors (Cannaert et al., 2017). Availability of such a method can potentially be used to screen biofluids for the presence of SCRAs without the chemical structural information. The assay can also be used to evaluate the “intrinsic potency” of SCRA formulations, as demonstrated by Antonides et al. who synthesized enantiospecifically 4 indazole-3-carboxamide-type SCRA for aiding their detection in seized drug samples.

Steuer et al. reviewed the recent development of metabolomics approaches for drugs of abuse testing based on the monitoring of changes in small endogenous molecules in response to drugs of abuse consumption and to specimen manipulation. Based on the limited number of studies reported in the literature, the authors conclude that metabolomic approaches possess potential for detection of biomarkers indicating drug consumption. More studies, including more sensitive targeted analyses, multi-variant statistical models or deep-learning approaches are needed to fully explore the potential of omics science in drugs of abuse testing, especially in testing consumption of unknown NPS.

Adding to the challenge of detecting the use of a large number of NPS available in the illicit drug market is the limited understanding of their biotransformation following consumption. Many NPS such as SCRAs undergo extensive metabolic transformation in the body and drug monitoring needs to target not only the parent drugs but also their metabolites. Studying the metabolism of these compounds is also important to better understand the toxicity of NPS as many NPS metabolites are also pharmacologically active and contribute to the overall toxicity of the parent drugs. Xu et al. studied the metabolism of AMB-FUBINACA, another indazole-3-carboxamide-type SCRA, using both human liver microsomes and zebra fish systems, and discovered a number of unique metabolites to be used as potential poisoning markers for drug monitoring purpose. Diao and Huestis reviewed the advantages and disadvantages of multiple metabolic approaches for investigating SCRA metabolism, including the human hepatocyte incubation model, human liver microsomes incubation, *in silico* prediction, rat *in vivo*, zebrafish, and fungus *Cunninghamella elegans* models and recommended the use of the human hepatocyte model when possible.

Another important aspect of the collection covers the development of sensitive analytical test methods for the detection of drugs of abuse in a variety of biological matrices. Chen et al. reported the development and validation of a simple, rapid, and sensitive LC-MS/MS method for chiral analysis of selegiline and its metabolites desmethylselegiline, R/S-methamphetamine, and R/S-amphetamine in dried urine spots. Busardò et al. developed a comprehensive LC-MS/MS method for quantifying fentanyl and 22 analogs and metabolites in whole blood, urine, and hair.

In summary, this collection covers Research Topics representative of a number of important research focuses in the area of drugs of abuse testing. Speedy, sensitive and accurate detection of drugs of abuse, including NPS, is part of the crucial steps in the fight against substance misuse and its associated crimes and harms.

## Author Contributions

All authors listed have made a substantial, direct and intellectual contribution to the work, and approved it for publication.

### Conflict of Interest Statement

SE was employed by company Elliott Forensic Consulting. The remaining authors declare that the research was conducted in the absence of any commercial or financial relationships that could be construed as a potential conflict of interest.
